# Supplementary material for: Integrating Acupuncture and Herbal Medicine into Assisted Reproductive Technology: A Systematic Review and Meta-Analysis of East Asian Traditional Medicine
Source: Healthcare (Basel). 2025 Jun 3;13(11):1326. doi: 10.3390/healthcare13111326 (PMC12155192; doi:10.3390/healthcare13111326)
Supplement: Supplementary file 1 [file healthcare-13-01326-s001.zip › healthcare-3625384-supplementary/Supplementary materials/S 1 Search Strategy.doc]

Supplementary table S 1: The Search Strategy of English Electronic Databases.

**Medline**

#10 #5 AND #8 AND #9: 1,553 results

#9 (((((((((randomized controlled trial[Publication Type]) OR controlled clinical trial[Publication Type]) OR randomized[Title/Abstract]) OR placebo[Title/Abstract]) OR drug therapy[MeSH Subheading]) OR randomly[Title/Abstract]) OR trial[Title/Abstract]) OR groups[Title/Abstract])) NOT ((animals[MeSH Terms]) NOT humans[MeSH Terms]) : 4,987,798 results

#8 #6 OR #7 : 478,988 results

#7 In Vitro Fertilizatio*[Title/Abstract] OR Test-Tube Fertilization[Title/Abstract] OR Fertilizatio* AND , Test-Tube[Title/Abstract] OR Test Tube Fertilizatio*[Title/Abstract] OR Fertilizations in Vitro[Title/Abstract] OR Test-Tube Babies[Title/Abstract] OR Bab* AND , Test-Tube[Title/Abstract] OR Test Tube Bab*[Title/Abstract] OR IVF[Title/Abstract] OR fertilization[Title/Abstract] OR Fertilization in Vitro[Title/Abstract] OR Sperm Injections, Intracytoplasmic[Title/Abstract] OR Injectio* AND , Intracytoplasmic Sperm[Title/Abstract] OR Intracytoplasmic Sperm Injectio*[Title/Abstract] OR Sperm Injection, Intracytoplasmic[Title/Abstract] OR ICSI[Title/Abstract] OR Injections, Sperm, Intracytoplasmic[Title/Abstract] OR oocytes[Title/Abstract] OR In Vitro Oocyte Maturation Techniques[Title/Abstract] OR egg collection[Title/Abstract] OR Oocyte[Title/Abstract] OR Ovocyt*[Title/Abstract] OR Oocyte In Vitro Maturation[Title/Abstract] OR In Vitro Oocyte Maturation[Title/Abstract] OR In Vitro Maturation of Oocytes[Title/Abstract] OR Reproductive Techniqu* AND , Assisted[Title/Abstract] OR Assisted Reproductive Techniqu*[Title/Abstract] OR Techniqu* AND , Assisted Reproductive[Transliterated Title] OR Assisted Reproductive Techni*[Title/Abstract] OR Reproductive Techni* AND , Assisted[Transliterated Title] OR Techni* AND , Assisted Reproductive[Title/Abstract] OR Reproductive Technolog* AND , Assisted[Title/Abstract] OR Technolog* AND , Assisted Reproductive[Title/Abstract] OR ART[Title/Abstract] OR Reproductive techniqu*[Title/Abstract] OR Techniqu* AND , Reproductive[Title/Abstract] OR Reproduction Techniqu*[Title/Abstract] OR Technique, Reproduction[Title/Abstract] OR Techniqu* AND , Reproduction[Title/Abstract] OR Reproduction Techni*[Title/Abstract] OR Techni* AND , Reproduction[Title/Abstract] OR Technolog* AND , Reproductive[Title/Abstract] OR Reproductive Technolog*[Title/Abstract] OR Embryo Transfe*[Title/Abstract] OR Transfe* AND , Embryo[Title/Abstract] OR Blastocyst Transfer[Title/Abstract] OR Tubal Embryo Transfer[Title/Abstract] OR Tubal Embryo Stage Transfer[Title/Abstract] OR Single Embryo Transfer[Title/Abstract] OR Embryo Transfe* AND , Single[Title/Abstract] OR Single Embryo Transfers[Title/Abstract] OR Transfe* AND , Single Embryo[Title/Abstract] OR implantation[Title/Abstract] OR Infertility[Title/Abstract] OR Sterility, Reproductive[Title/Abstract] OR Sterility[Title/Abstract] OR Reproductive Sterility[Title/Abstract] OR Subfertility[Title/Abstract] OR Sub-Fertility[Title/Abstract] OR Infertility, Female[Title/Abstract] OR Female Infertility[Title/Abstract] OR Sterility, Postpartum[Title/Abstract] OR Postpartum Sterility[Title/Abstract] OR Subfertility, Female[Title/Abstract] OR Female Subfertility[Title/Abstract] OR Sub-Fertility, Female[Title/Abstract] OR Female Sub-Fertility[Title/Abstract] OR Sub Fertility, Female[Title/Abstract] OR Sterility, Female[Title/Abstract] OR Female Sterility[Title/Abstract]: 282,558 results

#6 "Fertilization in Vitro"[Mesh] OR "Sperm Injections, Intracytoplasmic"[Mesh] OR "Oocytes"[Mesh] OR "In Vitro Oocyte Maturation Techniques"[Mesh] OR "Reproductive Techniques, Assisted"[Mesh] OR "Reproductive Techniques"[Mesh] OR "Embryo Transfer"[Mesh] OR "Single Embryo Transfer"[Mesh] OR "Infertility, Female"[Mesh] OR "Infertility"[Mesh]: 255,406 results

#5 #1 OR #2 OR #3 OR #4: 455,723 results

#4 "herbal Medicine / methods*"[Title/Abstract] OR "Medicine, Kanpo*"[Title/Abstract] OR "Kanpo Medicine*"[Title/Abstract] OR "Kampo*"[Title/Abstract] OR "Kanpo*"[Title/Abstract] OR "traditional Chinese Medicine*"[Title/Abstract] OR "Chinese Medicine, Traditional*"[Title/Abstract] OR "traditional Medicine, Chinese*"[Title/Abstract] OR “Drug Safety*"[Title/Abstract] OR “Medicine, Complementary*"[Title/Abstract] OR “Complementary Medicine*"[Title/Abstract] OR “Medicine, Alternative*"[Title/Abstract] OR “Therapy, Alternative*"[Title/Abstract] OR “Drugs, Chinese Herbal*"[Title/Abstract] OR “Herbal Drugs, Chinese*"[Title/Abstract] OR “Plant Extracts, Chinese*"[Title/Abstract] OR “Chinese Herbal Drugs*"[Title/Abstract] OR “Medicine, Herbal*"[Title/Abstract] OR “Herbal Therapy *"[Title/Abstract] OR “Herb Therapy*"[Title/Abstract] OR “Traditional Medicine, Korean*"[Title/Abstract] OR “Korean Traditional Medicine*"[Title/Abstract]: 47,042 results

#3 "Medicine, East Asian Traditional" [Mesh] OR "Herbal Medicine" [Mesh] OR "Medicine, Kampo" [Mesh] OR "Medicine, Chinese Traditional" [Mesh] OR "Complementary Therapies"[Mesh] OR "Medicine, Chinese Traditional"[Mesh] OR "Drugs, Chinese Herbal"[Mesh] OR "Phytotherapy"[Mesh] OR "Medicine, Korean Traditional"[Mesh]: 283,905 results

#2 acupuncture[Title/Abstract] AND moxibustion[Title/Abstract] OR auricular-acupuncture[Title/Abstract] OR laser acupuncture[Title/Abstract] OR transcutaneous electrical acupoint stimulation[Title/Abstract] OR TEAS[Title/Abstract] OR Needl*[Title/Abstract] OR acupuncture[Title/Abstract] OR acupuncture therapy[Title/Abstract] OR moxibustion[Title/Abstract] OR electroacupuncture[Title/Abstract] OR Acupuncture Points[Title/Abstract] OR acupressure[Title/Abstract] OR Acupuncture, Ear[Title/Abstract] OR auriculotherapy[Title/Abstract] OR meridians[Title/Abstract] OR Auriculotherapies[Title/Abstract] OR Acupunctures, Ear[Title/Abstract] OR Ear Acupunctures[Title/Abstract] OR Acupuncture, Auricular[Title/Abstract] OR Acupunctures, Auricular[Title/Abstract] OR Auricular Acupunctures[Title/Abstract] OR Pharmacopuncture[Title/Abstract] OR Acupuncture Treatmen*[Title/Abstract] OR Treatment, Acupuncture[Title/Abstract] OR Therapy, Acupuncture[Title/Abstract] OR Treatment, Pharmacoacupuncture[Title/Abstract] OR Pharmacoacupuncture Therapy[Title/Abstract] OR Therapy, Pharmacoacupuncture[Title/Abstract] OR Acupotomy[Title/Abstract] OR Acupotomies[Title/Abstract] OR Acupuncture Point[Title/Abstract] OR Point, Acupuncture[Title/Abstract] OR Points, Acupuncture[Title/Abstract] OR Acupoints[Title/Abstract] OR Acupoint[Title/Abstract]: 172,103 results

#1 "Acupuncture"[Mesh] OR "Acupuncture Therapy"[Mesh] OR "Moxibustion"[Mesh] OR "Electroacupuncture"[Mesh] OR "Acupuncture Points"[Mesh] OR "Acupressure"[Mesh] OR "Acupuncture, Ear"[Mesh] OR "Auriculotherapy"[Mesh] OR "Meridians"[Mesh] OR “Needles”[Mesh]: 46,462 results

**Cochrane library**

#1 Acupuncture

#2 Acupuncture Therapy

#3 Auriculotherapy

#4 Moxibustion

#5 Meridians

#6 Needles

#7 Electroacupuncture

#8 Herbal Medicine

#9 Kampo Medicine

#10 Traditional Chinese Medicine

#11 #1 OR #2 OR #3 OR #4 OR #5 OR #6 OR #7 OR #8 OR #9 OR #10

#12 (acupunct*):ti,ab,kw OR (acupoint*):ti,ab,kw OR (electroacupunct*):ti,ab,kw OR (electro‐acupunct*):ti,ab,kw OR (auriculotherap*):ti,ab,kw

#13 (auriculoacupunct*):ti,ab,kw OR (moxibust*):ti,ab,kw OR (scalptherap*):ti,ab,kw OR (scalpacupunct*):ti,ab,kw

#14 #11 OR #12 OR #13

#15 Fertilization in Vitro

#16 Sperm Injections, Intracytoplasmic

#17 Reproductive Techniques, Assisted

#18 Embryo Transfer

#19 #15 OR #16 OR #17 OR #18

#20 (randomized):ti,ab,kw OR (randomised):ti,ab,kw OR (RCT):ti,ab,kw OR (random*):ti,ab,kw OR (trial):ti,ab,kw

#21 Randomized Controlled Trial

#22 #20 OR #21

#23 #14 AND #19 AND #22

**Embase**

#23 #14 AND #19 AND #22

#22 #20 OR #21

#21 Randomized AND Controlled AND Trial

#20 (randomized):ti,ab,kw OR (randomised):ti,ab,kw OR (RCT):ti,ab,kw OR (random*):ti,ab,kw OR (trial):ti,ab,kw

#19 #15 OR #16 OR #17 OR #18

#18 Embryo AND Transfer

#17 Reproductive AND Techniques, AND Assisted

#16 Sperm AND Injections, AND Intracytoplasmic

#15 Fertilization AND in AND Vitro

#14 #11 OR #12 OR #13

#13 (auriculoacupunct*):ti,ab,kw OR (moxibust*):ti,ab,kw OR (scalptherap*):ti,ab,kw OR (scalpacupunct*):ti,ab,kw

#12 (acupunct*):ti,ab,kw OR (acupoint*):ti,ab,kw OR (electroacupunct*):ti,ab,kw OR (electro‐acupunct*):ti,ab,kw OR (auriculotherap*):ti,ab,kw

#11 #1 OR #2 OR #3 OR #4 OR #5 OR #6 OR #7 OR #8 OR #9 OR #10

#10 Traditional AND Chinese AND Medicine

#9 Kampo AND Medicine

#8 Herbal AND Medicine

#7 Electroacupuncture

#6 Needles

#5 Meridians

#4 Moxibustion

#3Auriculotherapy

#2Acupuncture AND Therapy

#1 ‘Acupuncture’/exp or acupuncture

**Web of science**

#1 Acupuncture OR Acupuncture Therapy OR Auriculotherapy　OR　Moxibustion　 OR　Meridians OR　Needles　 OR　Electroacupuncture　 OR　Herbal Medicine　 OR　Kampo Medicine　 OR　Traditional Chinese Medicine : 325,276

#2 Fertilization in Vitro OR Sperm Injections, Intracytoplasmic OR Reproductive Techniques, Assisted OR Embryo Transfer

#3 #1 AND #2 : 82,005

#4 Randomized Controlled Trial: 509.515

#5 #3 AND #4: 170
