# Supplementary material for: Integrating Acupuncture and Herbal Medicine into Assisted Reproductive Technology: A Systematic Review and Meta-Analysis of East Asian Traditional Medicine
Source: Healthcare (Basel). 2025 Jun 3;13(11):1326. doi: 10.3390/healthcare13111326 (PMC12155192; doi:10.3390/healthcare13111326)
Supplement: Supplementary file 1 [file healthcare-13-01326-s001.zip › healthcare-3625384-supplementary/Supplementary materials/S 2 Study characteristics.doc]

**Supplementary tables S2 study characteristics**

**Table 1: Acupuncture study design, intervention’s details, and outcome of selected RCT studies (Total: 25)**

| Study  Year  [Reference]0 | Country | Participants | | Mean age ±SD or  (range, year) | | Intervention type, timing and sessions | | Main IVF  Outcomes |
| --- | --- | --- | --- | --- | --- | --- | --- | --- |
|  |  | IG | CG | IG | CG | IG | CG |  |
| Dong et al.  2023 | China | 34 | 35 | 29.5±3.50 | 29.75±3.72 | Traditional Ac +IVF-ET  Timing: during IVF  4 sessions: once a day for 4 days from the day of OA to the day of ET. 30 min each.  Acupoints: CV3, CV6, SP6, LR3, ST25, ST29 and ST36. | Sham Ac +IVF-ET  Sessions and timing as IG.  Non-acupoints on each shoulder and upper arm.. | IR  CPR:IG>CG  LBR:IG>CG  AE  AR |
| Wu et al.  2022 | China | 40 | 43 | 29.18±5.39 | 29.51±2.96 | Ac+IVF-ET  Timing: before IVF  7-8 sessions: once every other day until the ovulation day, 30 min each.  Acupoints: Group 1:CV3, CV4, CV6, EX-CA1; Group 2: GV4, GV3, CV3, BL23, BL32. | IVF GnRH agonist long protocol | CPR:IG>CG LBR:IG>CG |
| Feng et al.  2022 | China | 334 | 330 | 31.5±4.4 | 31.7±4.7 | TEAS  Timing: during IVF  2 sessions: 24 h before ET and 30 min after ET, 30 min each.  Acupoints: Before ET: SP8, ST29, NR19 (Zigong), and SP10. After ET: ST36, KI3, BL23, RN4, and RN12. | IVF‑ET Protocol | CPR:IG>CG LBR:IG>CG BPR  IR  AR |
| Zhai et al.  2022  Five-Armed | China | 40  40  40  40 | 40 | 32.05±3.71  31.03±3.06  30.68±2.95  30.98±3.09 | 31.28±3.56 | TEAS groups: E-I, E-II, E-III, and E-IV  Timing: during IVF  10-12 sessions: from Day 3 to the day of HCG, once a day, 30 min each.  Acupoints: RN4, RN3, SP6, EX-CA1 (Zigong), and KI13. | Mock TEAS  Same as IG. | CPR:IG>CG GQE |
| Dehghani et al.  2020  Three-armed | Iran | 62  62 | 62 | 32.9±4.8  32.1±5.9 | 31.5±5.4 | ACU1 group  1 session: 25 min before ET  ACU 2 group  2 sessions: 25 min before and after ET  Timing: during IVF  Acupoints: HT7, PC6, Ren6, DU20, SP6, and Ren4. | GnRH agonist long protocol | CPR:IG>CG BPR  OPR |
| Guven et al.  2019 | Turkey | 36 | 36 | 30.3±3.4 | 31.5±4.0 | IVF‑ET +Ac  Timing: during IVF  3 sessions: One week before ET, 30 min before and after ET, 30 min each.  Acupoints: 1^st^ session: HT7, LI4, GV20, ear shenmen; 2^nd^ session: CV3, CV4, CV6, GV20, LIV3, ST30, and SP8; 3^rd^ session: LI4, SP6, SP9, ST36. | IVF‑ET Protocol | CPR:IG>CG LBR:IG>CG OPR |
| Altutunji et al.  2019 | China | 33 | 69 | 20-40 | 20-40 | Acupuncture  Timing: during IVF  2 weeks sessions: Starting from Day 3 daily till the day of giving HCG, 30-40 min each.  Acupoints: LIV3, SP6, SP8, ST36, SP10, ST29, LI4, Ren4. | IVF‑ET Protocol | CPR:IG>CG OPR  OHSS  EPR  NET  BPR |
| Shuai et al.  2019 | China | 61 | 61 | 31.23±3.78 | 31.58±3.07 | IVF+TEAS  Timing: during IVF  10 sessions: from Day 5 of the ovarian stimulation cycle once every other day until the day of ET, 30 min each.  Acupoints: SP6, CV3, CV4 and EX-CA1 (Zigong). | IVF+Mock TEAS  Same as IG. | CPR:IG>CG LBR:IG>CG IR |
| Gillerman et al.  2018 | UK | 64 | 69 | 32.88±17.16 | 34.93±4.142 | Ac+ IVF  Timing: during IVF  3 sessions: 25 min each  1^st^ session: between days 6 and 8 of the stimulated cycle. 25 min  2^nd^ and 3^rd^ session: on the day of ET − before and immediately after ET.  Acupoints: ST29, CV4, CV6, SP6, SP10; Before ET: SP8, SP10, LR3, ST29, CV4 and one selected from T7/PC6/YinTang. Ear Shenmen and Zigong were used on the day of ET. After ET: GV20, KD3, ST36, SP6, PC6. | GnRH agonist long protocol | CPR:IG>CG LBR:IG>CG  MR |
| Smith et al.  2018 | Australia New Zealand | 424 | 424 | 35.4±4.3 | 35.5±4.3 | Ac+IVF  Timing: during IVF  3 sessions: the first undertaken on day 9 of stimulating injections, the second before ET, and the third immediately after ET. 25 min each  Acupoints:  1^st^ session: ST29, Ren4, Ren6, SP6, and SP10. 2^nd^ and 3^rd^ session: within 1 hour prior to ET: ST29, SP8, SP10, LR3, Ren4; 1 point from HT7, PC6, or YinTang; and auricular point Zhigong. 3^rd^ session following ET, DU20, KD3, ST36, SP6, PC6, and auricular point ShenMen. | Sham Ac (noninsertive) +IVF  Same sessions and acupoints as IG. | CPR:IG>CG LBR:IG>CG MR  AE (Tiredness, Dizziness, Pain, Bruising, Nausea, Headaches, Diarrhea) |
| Qu et al.  2017  Four-Armed | China | 108  111  114 | 109 | 31.22±5.92  30.44±5.82  31.16±6.09 | 29.81±6.17 | TEAS-2 Hz group  TEAS-100 Hz group  TEAS-2/100 Hz  Timing: during IVF  2 sessions: 24 h before TVOR and 2h before ET. 30 min each  Acupoints: SP10, SP8, LR3, ST36, EX-CA1 (Zigong), RN4, PC6 and RN12. | GnRH agonist long protocol | CPR:IG>CG LBR:IG>CG IR  NRO  GQE |
| Zheng et al. 2015  Four-Armed | China | 56 | 56  54  60 | 36.05±5.48 | 36.88±4.65  36.85±5.37  36.95±4.34 | TEAS (D)  Timing: before and during IVF  35 Sessions: start day 7, once a day, a total of three courses until the day of egg retrieval. 30 min each. Acupoints: SP6, CV3, CV4, GV3, GV4, ST25, BL23, EX-CA1 | False Han’s placebo (C)  Artificial endometrial cycle treatment (B)  IVF‑ET Protocol (A) | CPR:IG>CG NRO  HQE  NET  AR |
| Qu et al.  2014  Three-armed | China | 101 | 102  102 | 31.65±4.30 | 30.87±4.12  30.95±4.78 | AA+IVF  Timing: during IVF  6 days consecutively press the acupoints four times a day (08:00, 12:00, 16:00 and 20:00 h respectively) with 15 min each time by themselves.  AA points: TF4 (Shenmen), CO18 (Endocrine) and TF2 (Internal Genitals) | Sham-AA +IVF  Sham-AA group: CO17 (Triple Energizer), CO4 (Stomach) and CO7 (Large Intestine)  IVF‑ET Protocol | CPR:IG>CG LBR: IG<CG  IR |
| Villahermosa et al.  2013  Three-armed | Brazil | 28 | 28  28 | 36.0±2.7 | 36.2±2.2  36.4±2.1 | True acupuncture  Timing: during IVF  4 sessions: D1, D7, day before the day of ovarian puncture and on the day after the day of ET. 20 min each.  Acupoints: PC6, KI3, KI6, KI7, KI10, LR3, SP4, SP6, SP10, ST40, LU7, Zigong. | Sham acupuncture  Points: eight needles insertion was performed in the arm and thigh at non-acupuncture points.  IVF‑ET Protocol | CPR:IG>CG BPR  NRO  NET |
| Rashidi et al.  2013 | Iran | 31 | 31 | 31.03±4.82 | 32.10±4.68 | Acupuncture +IVF –ET/ICSI  Timing: during IVF  5 sessions: Start of down regulation, start of stimulation, 2 days before OPU, and before and after ET. 30 min each.  Acupoints: LI4, SP6, SP10, PC6, LR3, CV4, GV20, ST36, ST29 and auricular points (Ovary, Uterus, Shenmen) | IVF‑ET /ICSI (COH) | CPR:IG>CG OPR  BPR  NRO  MR |
| Zhang et al.  2011  Three-armed | China | 110  100 | 99 | 31.9±5.3  32.6±4.9 | 31.5±5.2 | Single TEAS (S.T.)  Timing: during IVF  1 session: 30 min after ET.  Acupoints: ST36 and KI3, BL23 and RN4  Double TEAS (D.T.)  2 sessions: 24 hours before ET and 30 min after ET. 30 min each.  Acupoints: SP8 and ST29, EX-CA1 (Zigong) and SP10. | Mock TEAS  One session: 30 min after ET.  Acupoints: same as the single TEAS group. | CPR:IG>CG LBR:IG>CG BPR  IR  PL |
| Moy et al.  2011 | USA | 87 | 74 | 33.30± 0.31 | 33.16± 0.33 | True acupuncture  Timing: during IVF  2 sessions: before and after ET, 25 min each.  Acupoints: Before ET: CV6, SP8, LIV3, ST29, DU20.  After ET: ST36, SP6, SP10, LI4.  AA (shenmen, sympathetic, uterus, endocrine). | Sham acupuncture  Acupoints: same as T.G, except AA (knee, heel, allergic area, mouth). | CPR:IG<CG |
| Andersen et al.  2010 | Denmark | 314 | 321 | 31 (26, 37) | 31 (26, 36) | Real acupuncture  Timing: during IVF  2 sessions: before and after ET, 30 min each.  Acupoints: Before ET: DU20, ST29, PC6, SP8 and LIV3. After ET: DU20, LI4, SP10, ST36 and SP6. | Placebo acupuncture  Same as IG. | CPR:IG<CG  LBR:IG<CG  OPR  IR |
| Wing Sze So et al.  2010 | China  Hong Kong | 113 | 113 | 36.0 (34–38) | 36.0 (34–39) | Real acupuncture  Timing: during IVF  1 session after FET: 25 min  Acupoints: ST36, SP6, SP10, and LI4. | Placebo acupuncture  Same as IG. | CPR:IG<CG  LBR:IG<CG  OPR  IR, MR |
| So et al.  2009 | China Hong  Kong | 185 | 185 | 36.0 (33–38) | 36.0 (34–38) | Real ac +IVF long protocol  Timing: during IVF  2 sessions: 25 min before and after ET, 25 min each.  Acupoints:  Before ET: PC6, SP8, LR3, GV20 and ST29. After ET: ST36, SP6, SP10 and LI4. | Placebo acu +IVF long protocol  Same as IG | CPR:IG<CG  LBR:IG<CG  OPR  IR  MR  EPR |
| Dieterle et al.  2006 | Germany | 116 | 109 | 35.1±3.8 | 34.7±4.0 | Real acupuncture  Timing: during IVF  2 sessions: 30 min after ET and 3 days later, 30 min each.  Acupoints:  After ET: RN4, RN6, ST29, PC6, SP10), SP8 + ear points: Shenmen, Zigong, Neifenmi, Pizhixia. Three days after ET: LI4, SP6, ST36, KI3, LR3 + Same ear points. | Placebo acupuncture  Two sessions: same as IG.  Acupoints:  After ET and again 3 days later: SJ9, SJ12, GB31, GB32, and GB34 + ear points: Shangzhi, Feng si, Sisheng, and Jian. | CPR:IG>CG OPR  IR  MR |
| Westergaard et al.  2006  Three-armed | Denmark | 95  91 | 87 | 37 (24–45)  37 (27–45) | 37 (27–45) | ET +ACU1  Timing: during IVF  2 sessions: on the day of ET (before and after), 25 min each.  Acupoints: Before ET: DU20, ST29, SP8, PC6, and LR3. After ET: ST36, SP6, SP10, and LI4.  ET+ACU2  3 sessions: 2 on day of ET and 1 on 2 days later, 25 min each.  Acupoints: On the day of ET: same as ACU 1. 2 days after ET: DU20, Ren3, ST29, SP10, SP6, ST36, and LI4. | IVF‑ET Protocol | CPR:IG>CG LBR:IG>CG OPR  IR  PL |
| Smith et al.  2006 | Australia | 110 | 118 | 35.4±4.3 | 35.5±4.3 | IVF+ True Ac  Timing: during IVF  3 sessions: 1 was days 9 of ovarian stimulation. 2 were given on the day of ET.  Acupoints: same as Paulus et al. 2002, except Liv3 and DU20. | Sham Ac (placebo needle)  Sham needles placed close to acupuncture points did not penetrate skin.  3 sessions same as IG. | CPR:IG>CG OPR  IR  AE |
| Stener-Victorin et al.  2003 | Sweden | 141 | 145 | 32.9 (22-38) | 32.9(25-38) | EA group (EA plus a PCB)  1 session: 30 min before oocyte aspiration  Timing: during IVF  Acupoints: ST36, GV20, ST29, TE5 and LI4. | Alfentanil group (alfentanil plus a PCB) | CPR: IG<CG  OPR  IR  MR  NET |
| Paulus et al.  2002 | Germany | 80 | 80 | 32.8±4.1 | 32.1±3.9 | ET+Ac  Timing: during IVF  2 sessions: Before and after ET. 25 min each.  Acupoints: Before ET: PC6, SP8, LIV3, GV20, and ST29. After ET: ST36, SP6, SP10, and LI4. | IVF‑ET Protocol | CPR:IG>CG |

Abbreviations: IG, intervention group; CG, control group; RCT, Randomized controlled trial; AA, auricular acupressure; Ac, ACU, acupuncture; AE, adverse events; AR, abortion rate; OA, oocyte aspiration; COS, Controlled ovarian stimulation; EA, electro-acupuncture; HCG, Human chorionic gonadotropin; PR, pregnancy rate; PL, pregnancy loss; IR, implantation rate; BPR, Biochemical pregnancy rate; COH, Controlled ovulation hyperstimulation; CPR, Clinical pregnancy rate; OPR, ongoing pregnancy rate; MR, Miscarriage rate; LBR, live birth rate; NET, number of embryos transferred; NOF, Number of ovarian follicles; NRO, Number of retrieved ova/ oocytes; NOM, number of oocyte maturation; GQE, good-quality embryos; NFO, number of fertilized oocytes; FR, Fertilization rate; ET, embryo transfer; EIR, embryo implantation rate; EPR, Ectopic pregnancy rate; FET, frozen embryo transfer; TEAS, transcutaneous electrical acupoint stimulation; TVOR, trans-vaginal oocyte retrieval; OPU, ovum pick-up; PCB, a paracervical block; OHSS, ovarian hyper stimulation syndrome; BL, Bladder meridian; CV, Conception Vessel meridian; GV, Governor vessel meridian; HT, Heart meridian; KD, KI, Kidney meridian; LI, large intestine meridian; LR, LIV, liver meridian; PC, Pericardium meridian; SP, Spleen meridian; ST, Stomach meridian; TE, Triple Warmer meridian; RN, Ren mai meridian. DU, Du mai meridian.

**Table 2: Herbal medicine study design, intervention’s details, and outcome of selected RCT studies (Total: 12)**

| Study ID | Country | Participants | | Age | | Intervention Formulae, dosage and duration | | Main Outcome |
| --- | --- | --- | --- | --- | --- | --- | --- | --- |
|  |  | IG | CG | IG | CG | IG | CG |  |
| Li et al.  2023 | China | 60 | 60 | 33.55±3.99 | 33.87±3.50 | Zishen Yutai Pills (ZYPs) + IVF(GnRH antagonist protocol)  Timing: before, during IVF and after ET  Duration: ZYPs 5 g, 3 times daily orally from the mid-luteal phase prior to the IVF cycle for nearly 20 days and lasted until the 12th week of gestation. | IVF(GnRH antagonist protocol) | CPR:IG=CG  LBR:IG=CG  IR  NRO  HQE  PL  EP |
| Ma et al.  2023  Three-armed | China | 38 | 37  39 | 36.87±3.12 | 37.49±3.19  29.23±2.79 | Guilu Erxian ointment + GnRH antagonist protocol  Timing: before IVF  Duration: Orally, 5 ml each time, 3 times a day, was administered on the third day of menstruation in the menstrual cycle before IVF for 40 days. | Placebo +IVF  Same as IG  IVF-ET/ICSI alone. | CPR:IG<CG  NRO  GQE  TNAM |
| Chen et al.  2022 | China | 1131 | 1134 | 30.7±4.2 | 30.7±4.1 | Zishen Yutai Pill (ZYP) +IVF  Timing: before, during IVF and after ET  Duration: orally, 5 g each time, three times per day, from day 19-23 in a previous cycle until the day of the pregnancy test (2 weeks after ET). | Placebo +IVF (long protocol, GnRH antagonist protocol)  Same as IG. | CPR:IG>CG LBR:IG>CG IR  BPR  PL |
| Sun et al.  2021 | China | 48 | 49 | 38.92±4.43 | 37.61±4.22 | Erzhi Tiangui (EZTG) granule +IVF  Timing: during IVF  Duration: orally, 3 g each time, 2 times a day, started on the 2nd to 3rd day of menstruation for 11–14 days. | Placebo+IVF  Same as IG. | CPR:IG>CG NRO  GQE |
| Song et al.  2021 | China | 231 | 231 | 37.9±2.3 | 37.8±2.2 | Ding-Kun Pill (DKP) +IVF  Timing: before and during IVF  Duration: orally, 7 g each time, twice daily on the previous menstrual cycle day 5 until oocyte retrieval, 5 to 6 weeks. | Placebo +IVF  Same as IG | CPR:IG>CG OPR  NRO  HQE  PL  EP |
| Cao et al.  2020 | China | 136 | 135 | 32.34±4.53 | 31.74±4.70 | Gushen’antai pills (GSATP) +FET  Timing: before, during IVF and after ET  Duration: orally, 6g, thrice a day, from day 1 of endometrial transformation until week 12 of pregnancy. The duration is 69 or 71 days. | Placebo +FET  Same as IG | CPR:IG>CG OPR  IR  AR  EPR  AE |
| Jiang et al.  2020 | China | 105 | 106 | 32.7±4.6 | 33.6±4.0 | Bushen Yutai+IVF (mild ovarian stimulation)  Timing: during IVF  Duration and dosage: N/A | IVF (mild ovarian stimulation) | CPR:IG>CG IR  MR  FETR |
| Xue et al.  2017 | China | 20 | 20 | 35.10±7.39 | 34.80±7.53 | Tiaogeng Yijing decoction (TGYJ) +IVF-ET  Timing: before IVF  Duration: Orally, 200 ml each time, twice a day, from day 7 of menstrual cycle for 2 weeks, repeated 3 cycles before IVF. | IVF-ET microstimulation | CPR:IG>CG NRO  HQE  HQO |
| Zhang et al.  2015  Three-armed | China | 56 | 57  55 | 34.57±4.15 | 33.75±5.36  34.72±4.77 | CM+FET  CM: Bushen Jianpi Recipe, Erzhi Pill, Shoutai Pill  Timing: before and after FET.  Duration: 60 days before and after FET.  Dosage: N/A. | Hysteroscopy group  FET Group | CPR:IG>CG BPR  IR |
| Guo et al. 2014 | China | 216 | 217 | 32.1±4.3 | 31.6±4.3 | CM+IVF  Timing: during IVF  Duration: Orally, 200 ml each time, twice a day, from day 8 of menstruation for 5 consecutive days. For a further 7-10 day after ET without the Danshen.  CM: Erzhi pill and Siwu decoction, Wenshenantai decoction. | IVF(conventional control ) | CPR:IG>CG LBR:IG>CG BPR  HQE  OHSS |
| Lian et al.  2014 | China | 33 | 33 | 30.33±3.23 | 30.70±3.42 | Liuwei Dihuang Granule +IVF-ET  Timing: before IVF  Duration: 3 menstrual cycles before IVF  Dosage: N/A. | Placebo granule +IVF-ET (a long protocol)  Same as IG. | CPR:IG>CG HQE  HQO |
| Gao et al.  2013 | China | 30 | 28 | 29.1±2.5 | 29.7±2.0 | Xiaoyao powder +IVF-ET  Timing: during IVF  Duration: Orally, 150 ml each time, twice a day, applied at the same time as ovulation stimulation until the day of hCG injection. | IVF-ET (GnRHa/FSH/ hCG only) | CPR:IG>CG NRO  HQE |

Abbreviations: IG, intervention group; CG, control group; HM, Herbal medicine; TCM, Traditional Chinese Medicine; CM, Chinese Medicine; RCT, randomized controlled trial; PR, pregnancy rate; BPR, biochemical pregnancy rate; OPR, ongoing pregnancy rate; CPR, clinical pregnancy rate; IR, implantation rate; PLR, pregnancy loss rate; AE, adverse events; AR, abortion rate; LBR, live birth rate; ET, embryo transfer; FETR, Fresh embryo transfer rate; GnRH, gonadotropin-releasing hormone; NOM, number of oocyte maturation; NRO, number of retrieved oocytes; GQE, good-quality embryos; FR, fertilization rate; TNAM, total number of available embryos; HQO, high-quality oocytes; HQE, high-quality embryos; CD, Cycle Day; EP, Ectopic pregnancy; N/A, not applicable.
