# Supplementary material for: Integrating Acupuncture and Herbal Medicine into Assisted Reproductive Technology: A Systematic Review and Meta-Analysis of East Asian Traditional Medicine
Source: Healthcare (Basel). 2025 Jun 3;13(11):1326. doi: 10.3390/healthcare13111326 (PMC12155192; doi:10.3390/healthcare13111326)
Supplement: Supplementary file 1 [file healthcare-13-01326-s001.zip › healthcare-3625384-supplementary/Supplementary materials/S 3 Beggs test and Eggers test.doc]

**Supplementary Tables S3 Begg’s test and Egger’s test**

**CPR_Ac**

Begg's Test

adj. Kendall's Score (P-Q) =	-2

Std. Dev. of Score =	50.62

Number of Studies =	28

z =	-0.04

Pr > |z| =	0.968

z =	0.02 (continuity corrected)

Pr > |z| =	0.984 (continuity corrected)

Egger's test

------------------------------------------------------------------------------

Std_Eff |	Coef. Std. Err. t	P>|t|	[95% Conf. Interval]

-------------+----------------------------------------------------------------

slope |	.0581288	.0504513	1.15	0.260	-.0455753	.1618328

bias |	4.246145	2.379271	1.78	0.086	-.6445161	9.136806

**CPR_HM**

Begg's Test

adj. Kendall's Score (P-Q) =	0

Std. Dev. of Score =	14.58

Number of Studies =	12

z =	0.00

Pr > |z| =	1.000

z =	-0.07 (continuity corrected)

Pr > |z| =	1.000 (continuity corrected)

Egger's test

------------------------------------------------------------------------------

Std_Eff |	Coef. Std. Err. t	P>|t|	[95% Conf. Interval]

-------------+----------------------------------------------------------------

slope |	.128153	.0392398	3.27	0.008	.0407213	.2155846

bias |	.6929945	2.941901	0.24	0.819	-5.861969	7.247958

------------------------------------------------------------------------------

**LBR_Ac**

Begg's Test

adj. Kendall's Score (P-Q) =	25

Std. Dev. of Score =	20.21

Number of Studies =	15

z =	1.24

Pr > |z| =	0.216

z =	1.19 (continuity corrected)

Pr > |z| =	0.235 (continuity corrected)

Egger's test

------------------------------------------------------------------------------

Std_Eff |	Coef. Std. Err. t	P>|t|	[95% Conf. Interval]

-------------+----------------------------------------------------------------

slope |	-.022788	.0836258	-0.27	0.790	-.2034505	.1578745

bias |	5.760429	3.632942	1.59	0.137	-2.088065	13.60892

------------------------------------------------------------------------------

**AE_ Ac+ HM**

Tests for Publication Bias

Begg's Test

adj. Kendall's Score (P-Q) =	35

Std. Dev. of Score =	28.58

Number of Studies =	19

z =	1.22

Pr > |z| =	0.221

z =	1.19 (continuity corrected)

Pr > |z| =	0.234 (continuity corrected)

Egger's test

------------------------------------------------------------------------------

Std_Eff |	Coef. Std. Err. t	P>|t|	[95% Conf. Interval]

-------------+----------------------------------------------------------------

slope |	.1698712	.072114	2.36	0.031	.017724	.3220184

bias | -1.199513	1.207374	-0.99	0.334	-3.746849	1.347824

Dropout rate_ETAM

Begg's Test

adj. Kendall's Score (P-Q) =	-34

Std. Dev. of Score =	22.21

Number of Studies =	16

z =	-1.53

Pr > |z| =	0.126

z =	1.49 (continuity corrected)

Pr > |z| =	0.137 (continuity corrected)

Egger's test

------------------------------------------------------------------------------

Std_Eff |	Coef. Std. Err. t	P>|t|	[95% Conf. Interval]

-------------+----------------------------------------------------------------

slope |	2.508743	.1893317	13.25	0.000	2.102667	2.91482

bias | -.0550377	.2430841	-0.23	0.824	-.5764012	.4663259
