# Supplementary material for: Integrating Acupuncture and Herbal Medicine into Assisted Reproductive Technology: A Systematic Review and Meta-Analysis of East Asian Traditional Medicine
Source: Healthcare (Basel). 2025 Jun 3;13(11):1326. doi: 10.3390/healthcare13111326 (PMC12155192; doi:10.3390/healthcare13111326)
Supplement: Supplementary file 1 [file healthcare-13-01326-s001.zip › healthcare-3625384-supplementary/Supplementary materials/S 4 Quality of evidence by GRADE.doc]

**Supplementary Tables S 4:** Quality of evidence included RCTs by GRADE

| Outcomes | Included RCTs (participants) | Relative effect (95% CI) | Certainty assessment | | | | | Quality of evidence |
| --- | --- | --- | --- | --- | --- | --- | --- | --- |
|  |  |  | Risk of bias | Inconsistency | Indirectness | Imprecision | Publication bias |  |
| Ac | | | | | | | | |
| Clinical pregnancy rate | 6610 | 1.316 (1.171, 1.480) | Serious Limitations | No serious limitations | No serious limitations | Serious Limitations | Serious Limitations | ⊕⊕⊕◯ Moderate |
| Live birth rate | 4613 | 1.287(1.081, 1.533) | Serious Limitations | No serious limitations | No serious limitations | Serious Limitations | Serious Limitations | ⊕⊕⊕◯ Moderate |
| Embryo implantation rate | 4173 | 1.183 (1.028, 1.363) | Serious Limitations | No serious limitations | No serious limitations | Serious Limitations | Serious Limitations | ⊕⊕⊕◯ Moderate |
| HM | | | | | | | | |
| Clinical pregnancy rate | 4343 | 1.184( 1.017, 1.379) | Serious Limitations | No serious limitations | No serious limitations | Serious Limitations | Serious Limitations | ⊕⊕⊕◯ Moderate |
| Live birth rate | 2818 | 1.147(1.010, 1.303) | Serious Limitations | No serious limitations | No serious limitations | Serious Limitations | Serious Limitations | ⊕⊕⊕◯ Moderate |
| Embryo implantation rate | 3035 | 1.106(0.968, 1.264) | Serious Limitations | No serious limitations | No serious limitations | Serious Limitations | Serious Limitations | ⊕⊕⊕◯ Moderate |
