# Supplementary material for: Integrating Acupuncture and Herbal Medicine into Assisted Reproductive Technology: A Systematic Review and Meta-Analysis of East Asian Traditional Medicine
Source: Healthcare (Basel). 2025 Jun 3;13(11):1326. doi: 10.3390/healthcare13111326 (PMC12155192; doi:10.3390/healthcare13111326)
Supplement: Supplementary file 1 [file healthcare-13-01326-s001.zip › healthcare-3625384-supplementary/Supplementary materials/S 5 Details of AEs in the 37 studies.doc]

**Supplementary table S 5: Categories of Adverse Events (AEs)**

| Study ID | Pregnancy loss  (N =5) | Abortion rate  (N = 4) | Adverse events  (N = 4) | Miscarriage rate  (N = 8) | Ovarian hyper stimulation syndrome  (N = 2) | Ectopic pregnancy rate  (N = 5) |
| --- | --- | --- | --- | --- | --- | --- |
| **Acupuncture (N = 14)** | | | | | | |
| Dong et al. 2023 |  | **✓** | **✓** |  |  |  |
| Feng et al. 2022 |  | **✓** |  |  |  |  |
| Altutunji et al. 2019 |  |  |  |  | **✓** | **✓** |
| Smith et al. 2018 |  |  | **✓** | **✓** |  |  |
| Gillerman et al. 2018 |  |  |  | **✓** |  |  |
| Zheng et al. 2015 |  | **✓** |  |  |  |  |
| Rashidi et al. 2013 |  |  |  | **✓** |  |  |
| Zhang et al. 2011 | **✓** |  |  |  |  |  |
| So et al. 2010 |  |  |  | **✓** |  |  |
| So et al. 2009 |  |  |  | **✓** |  | **✓** |
| Dieterle et al. 2006 |  |  |  | **✓** |  |  |
| Westergaard et al. 2006 | **✓** |  |  |  |  |  |
| Smith et al. 2006 |  |  | **✓** |  |  |  |
| Stener-Victorin et al. 2003 |  |  |  | **✓** |  |  |
| **Herbal medicine (N = 6)** | | | | | | |
| Li et al. 2023 | **✓** |  |  |  |  | **✓** |
| Chen et al. 2022 | **✓** |  |  |  |  |  |
| Song et al. 2021 | **✓** |  |  |  |  | **✓** |
| Cao et al. 2020 |  | **✓** | **✓** |  |  | **✓** |
| Jiang et al. 2020 |  |  |  | **✓** |  |  |
| Guo et al. 2014 |  |  |  |  | **✓** |  |

**Supplementary table S 5: Outcome of Adverse Events (AEs)**

| **Categories of Intervention** | **Outcome Variables of adverse events** | **Number of study** |
| --- | --- | --- |
| Acupuncture (N = 14) | Abortion rate | **3(**Dong et al. 2023; Feng et al. 2022; Zheng et al. 2015) |
|  | Adverse events | **3 (**Dong et al. 2023; Smith et al. 2018; Smith et al. 2006*) |
|  | Ectopic pregnancy rate | **2 (**Altutunji et al. 2019; So et al. 2009) |
|  | Miscarriage rate | **7 (**Smith et al. 2018; Gillerman et al. 2018; Rashidi et al. 2013; So et al. 2010; So et al. 2009;Dieterle et al. 2006; Stener-Victorin et al. 2003) |
|  | Ovarian hyper stimulation syndrome | **1(**Altutunji et al. 2019) |
|  | Pregnancy loss | **2 (**Zhang et al. 2011; Westergaard et al. 2006) |
| Herbal medicine (N = 6) | Abortion rate | **1 (**Cao et al. 2020) |
|  | Adverse events | **1 (**Cao et al. 2020) |
|  | Ectopic pregnancy rate | **3 (**Li et al. 2023; Song et al. 2021; Cao et al. 2020) |
|  | Miscarriage rate | **1 (**Jiang et al. 2020) |
|  | Ovarian hyper stimulation syndrome | **1 (**Guo et al. 2014) |
|  | Pregnancy loss | **3 (**Li et al. 2023; Chen et al. 2022; Song et al. 2021) |
