# Supplementary material for: Integrating Acupuncture and Herbal Medicine into Assisted Reproductive Technology: A Systematic Review and Meta-Analysis of East Asian Traditional Medicine
Source: Healthcare (Basel). 2025 Jun 3;13(11):1326. doi: 10.3390/healthcare13111326 (PMC12155192; doi:10.3390/healthcare13111326)
Supplement: Supplementary file 1 [file healthcare-13-01326-s001.zip › healthcare-3625384-supplementary/Supplementary materials/S 6 Reasons for dropping out in the 37 studies.doc]

Supplementary table S 6. Reasons for dropping out in the 37 studies of RCTs.

| **Reasons for the Drop-outs** | **EATM Group** | **Control Group** |
| --- | --- | --- |
| Withdrawal of Consent | 5 | 3 |
| AE | 0 | 1 |
| SAE | 0 | 0 |
| Lost to Follow-up | 20 | 24 |
| Discontinued Intervention | 21 | 19 |
| Violation of Inclusion and Exclusion Criteria | 51 | 33 |
| Protocol Deviation | 7 | 3 |
| Drop off | 22 | 22 |
| Cancelled cycle | 25 | 31 |
| Other | 30 | 20 |
| **Total** | 181 | 156 |

AE: Adverse Events / SAE: Severe Adverse Events

EATM therapies, including acupuncture (classic acupuncture, electro-acupuncture, laser acupuncture, auricular acupuncture); and herbal medicine (herbal powder, pills, granule, decoction, ointment).
