# Supplementary material for: Integrating Acupuncture and Herbal Medicine into Assisted Reproductive Technology: A Systematic Review and Meta-Analysis of East Asian Traditional Medicine
Source: Healthcare (Basel). 2025 Jun 3;13(11):1326. doi: 10.3390/healthcare13111326 (PMC12155192; doi:10.3390/healthcare13111326)
Supplement: Supplementary file 1 [file healthcare-13-01326-s001.zip › healthcare-3625384-supplementary/Supplementary materials/S 7 Commonly used acupoints and meridians.doc]

**Supplementary /Table 7: Commonly used acupoints and meridians for acupuncture treatment in IVF patients.**

| **Acupuncture points** | **Frequency (N=time)** | **Meridian** | **Frequency (N=time)** |
| --- | --- | --- | --- |
| Sanyinjiao (SP6) | **19** | Spleen Meridian (SP) | **49** |
| Zusanli (ST36) | **17** | Stomach Meridian (ST) | **36** |
| Guilai (ST29) | **15** | Stomach Meridian | **36** |
| Xuehai (SP10) | **15** | Spleen Meridian | **49** |
| Diji (SP8) | **13** | Spleen Meridian | **49** |
| Taichong (LR3) | **13** | Liver Meridian (LR) | **13** |
| Guanyuan (CV/RN4) | **13** | Conception Vessel Meridian /Ren Meridian (CV/RN4) | **29** |
| Hegu (LI4) | **12** | Large Intestine Meridian (LI) | **12** |
| Neiguan (PC6) | **12** | Pericardium Meridian (PC) | **12** |
| Baihui (GV/DU20) | **11** | Governor Vessel Meridian/ Du mai Meridian (GV/DU) | **15** |
| Qihai (CV/RN6) | **8** | Conception Vessel Meridian /Ren Meridian | **29** |
| Zigong (EX-CA1) | **7** | Extraordinary Meridian(EX) | **7** |
| Zhongji (CV/RN3) | **6** | Conception Vessel Meridian /Ren Meridian | **29** |
| Taixi (KI3) | **6** | Kidney Meridian (KI) | **10** |
| Shenshu (BL23) | **4** | Bladder Meridian (BL) | **5** |
| Zhongwan (CV/RN12) | **2** | Conception Vessel Meridian /Ren Meridian | **29** |
| Yaoyangguan(GV3) | **2** | Governor Vessel Meridian (GV) | **15** |
| Mingmen(GV4) | **2** | Governor Vessel Meridian | **15** |
| Tianshu (ST25) | **2** | Stomach Meridian | **36** |
| Shenmen(HT7) | **2** | Heart Meridian | **2** |
| Yinlingquan(SP9) | **1** | Spleen Meridian | **49** |
| Zhaohai (KI6) | **1** | Kidney Meridian | **10** |
| Fuliu (KI7) | **1** | Kidney Meridian | **10** |
| Yingu(KI10) | **1** | Kidney Meridian | **10** |
| Qixue(KI13) | **1** | Kidney Meridian | **10** |
| Qichong(ST30) | **1** | Stomach Meridian | **36** |
| Fenglong(ST40) | **1** | Stomach Meridian | **36** |
| Ciliao (BL32) | **1** | Bladder Meridian | **5** |
| Waiguan (TE5) | **1** | Triple Energizer Meridian | **1** |
| Gongsun (SP4) | **1** | Spleen Meridian | **49** |
| Lie Que (LU7) | **1** | Lung Meridian | **1** |

*Thirty-one acupoints from 25 RCTs are included in the present pattern analysis.
